# Correlates of Iron, Cobalamin, Folate, and Vitamin A Status among Stunted Children: A Cross-Sectional Study in Uganda

**DOI:** 10.3390/nu15153429

**Published:** 2023-08-02

**Authors:** Rolland Mutumba, Hannah Pesu, Joseph Mbabazi, Eva Greibe, Mette F. Olsen, André Briend, Christian Mølgaard, Christian Ritz, Nicolette Nabukeera-Barungi, Ezekiel Mupere, Suzanne Filteau, Henrik Friis, Benedikte Grenov

**Affiliations:** 1 Department of Nutrition, Exercise and Sports, University of Copenhagen, 1958 Frederiksberg C, Denmark; hannahpesu@gmail.com (H.P.); mjosef2000@gmail.com (J.M.); mette.frahm.olsen@regionh.dk (M.F.O.); cm@nexs.ku.dk (C.M.); hfr@nexs.ku.dk (H.F.); bgr@nexs.ku.dk (B.G.); 2 Department of Paediatrics and Child Health, School of Medicine, College of Health Sciences, Makerere University, Kampala P.O. Box 7072, Uganda; nicolettebarungi@gmail.com (N.N.-B.); mupez@yahoo.com (E.M.); 3 Department of Clinical Biochemistry, Aarhus University Hospital, 8200 Aarhus N, Denmark; evagreibe@gmail.com; 4 Tampere Center for Child, Adolescent and Maternal Health Research, Faculty of Medicine and Health Technology, Tampere University and Tampere University Hospital, 33520 Tampere, Finland; andre.briend@gmail.com; 5 National Institute of Public Health, University of Southern Denmark, 5230 Odense, Denmark; ritz@sdu.dk; 6 Faculty of Epidemiology and Population Health, London School of Hygiene and Tropical Medicine, London WC1E 7HT, UK; suzanne.filteau@lshtm.ac.uk

**Keywords:** stunting, micronutrient status, iron, cobalamin, folate, vitamin A

## Abstract

Micronutrient deficiencies and stunting are prevalent. We assessed correlates of iron, cobalamin, folate, and vitamin A biomarkers in a cross-sectional study of stunted children aged 12–59 months in eastern Uganda. The biomarkers measured were serum ferritin (S-FE), soluble transferrin receptor (S-TfR), retinol binding protein (S-RBP), plasma cobalamin (P-Cob), methylmalonic acid (P-MMA), and folate (P-Fol). Using linear regression, we assessed socio-demography, stunting severity, malaria rapid test, and inflammation as correlates of micronutrient biomarkers. Of the 750 children, the mean (SD) age was 32.0 (11.7) months, and 45% were girls. Iron stores were depleted (inflammation-corrected S-FE < 12 µg/L) in 43%, and 62% had tissue iron deficiency (S-TfR > 8.3 mg/L). P-Cob was low (<148 pmol/L) and marginal (148–221 pmol/L) in 3% and 20%, and 16% had high P-MMA (>0.75 µmol/L). Inflammation-corrected S-RBP was low (<0.7 µmol/L) in 21% and P-Fol (<14 nmol/L) in 1%. Age 24–59 months was associated with higher S-FE and P-Fol and lower S-TfR. Breastfeeding beyond infancy was associated with lower iron status and cobalamin status, and malaria was associated with lower cobalamin status and tissue iron deficiency (higher S-TfR) despite iron sequestration in stores (higher S-FE). In conclusion, stunted children have iron, cobalamin, and vitamin A deficiencies. Interventions addressing stunting should target co-existing micronutrient deficiencies.

## 1. Introduction

Micronutrients, i.e., vitamins and some minerals needed in very small amounts, are essential for growth and optimal body functions [1]. In low- and middle-income countries, multiple micronutrient deficiencies often co-exist in the same individual, and biomarkers suggesting an insufficient status may be present even in the absence of overt signs. Globally, it is estimated that there are approximately 372 million children aged 6–59 months with one or more micronutrient deficiencies, of which a quarter (98 million) are found in sub-Saharan Africa [2]. Micronutrient deficiencies have been associated with multiple adverse health outcomes. Iron, cobalamin, folate, and vitamin A deficiencies can cause nutritional anaemia [3], and iron, cobalamin, and folate deficiencies are associated with impaired cognitive development [4,5,6]. Vitamin A deficiency is associated with morbidity and mortality risk due to diarrhoea and respiratory tract infections as well as visual impairment [7]. Overall, approximately 34–52% of children in Africa have iron deficiency [8]. In 2013, sub-Saharan Africa had the highest burden of vitamin A deficiency among children under five years of age [9]. A recent review reported that the prevalence of low and marginal cobalamin ranged from 33% to 69% in studies among African children [10].

Globally, two out of five children with stunting live in Africa [11]. Children with stunting are vulnerable to micronutrient deficiencies. Factors that lead to stunting, such as poor nutrition and frequent infection, can also lead to micronutrient deficiencies [12]. Notably, diets in sub-Saharan Africa are commonly low in animal-source foods [13], which are the only natural sources of cobalamin, highly bioavailable haem iron, and preformed vitamin A. Inflammation and infections affect the validity of some biomarkers of micronutrient status [14] but also result in poor intake, impaired absorption, and utilization of micronutrients [15]. In turn, micronutrient deficiencies may result in impaired immunity, creating a vicious cycle. Stunting is associated with poor child survival, poor cognitive development, and reduced working capacity later in life [12,16]. Micronutrient deficiencies, such as iron or cobalamin deficiency, could contribute to the poor cognitive development observed among stunted children. Stunting is considered difficult to reverse after two years [17]; however, there are interventions that can improve micronutrient status and possibly the consequences of micronutrient deficiencies associated with stunting. Therefore, it is important to understand the nature and severity of micronutrient deficiencies in stunted children.

We aimed to assess the levels and correlates of biomarkers reflecting iron, cobalamin, folate, and vitamin A status among Ugandan children aged 12–59 months with stunting.

## 2. Materials and Methods

### 2.1. Design and Setting

This cross-sectional study was based on baseline data from the MAGNUS trial (ISRCTN13093195), a randomized, controlled, 2 × 2 factorial trial assessing the effects of milk protein and whey permeate in lipid-based nutrient supplements (LNS) on growth in 750 children with stunting [18]. The study was conducted in 2020 in Jinja District, eastern Uganda, where the prevalence of child stunting was estimated to be 29% [19]. Two local community health centres in the Walukuba Division and Buwenge Town Council were used as study sites.

### 2.2. Participants and Recruitment

Communities within Jinja District were mobilized by village health teams for the initial screening. Within the communities, the study staff identified 12-to 59-month-old children with stunting based on length/height-for-age z-scores (HAZ) and referred them to sites for final eligibility assessment. Length was measured for children aged ≤ 24 months and height for those aged > 24 months. At the study sites, children between 12 and 59 months of age who were residents in the catchment area were recruited if they were identified as stunted (HAZ < −2) and the caregiver provided informed consent. Children were excluded if they were identified as having severe acute malnutrition (weight-for-length/height z-score (WHZ) < −3, a mid-upper arm circumference (MUAC) < 115 mm, or oedema), a medical complication requiring hospitalization, or a disability that impeded length/height measurement, as well as if a child from the same household was already included or if the family planned to leave the catchment area within 6 months. Children with obvious disabilities that impeded eating capacity or a history of allergy to peanuts or milk, and those participating in another study, were also excluded.

### 2.3. Data Collection and Measurements

Trained staff collected sociodemographic and anthropometric data. Length/height was measured to the nearest 0.1 cm using an infant/child ShorrBoard (Weigh and Measure, Olney, MD, USA). Weight was measured to the nearest 100 g using an electronic scale (SECA 876; Hamburg, Germany). MUAC was measured to the nearest 0.1 cm using a standard measuring tape (UNICEF SD, Copenhagen, Denmark) on the left arm. All anthropometric measurements were performed in triplicate by trained staff, and the median was used.

Venous blood (approximately 6.0 mL) was collected from the cubital vein and divided into plain serum tubes, lithium heparin tubes, and ethylenediaminetetraacetic acid (EDTA) tubes. At the field laboratory, malaria was diagnosed from EDTA whole blood using a rapid diagnostic test (SD BIOLINE MALARIA AG PF, Abbott, Lake Forest, IL, USA). Samples were transported for processing at room temperature (20–25 °C) in a closed box. The rest of the blood was processed as soon as possible and usually within 3–4 h into either serum or plasma by centrifugation at 3500 rpm for 10 min and then aliquoted into and stored in cryovials at −20 °C. On a weekly basis, the processed samples were transferred in a cold box to the IBRH3AU biorepository at Makerere University in Kampala for storage at −80 °C. The processed samples were transferred to Denmark and Germany on dry ice for the analysis of micronutrient biomarkers and acute phase proteins.

Serum ferritin (S-FE), serum soluble transferrin receptor (S-TfR), serum retinol binding protein (S-RBP), serum C-reactive protein (S-CRP), and serum α_1_ acid glycoprotein (S-AGP) were determined at the VitMin Lab in Willstaedt, Germany, using a combined sandwich enzyme-linked immunosorbent assay [20]. Inter- and intra-assay coefficients of variation were 5–14%. Plasma cobalamin (P-Cob), plasma methylmalonic acid (P-MMA), and plasma folate (P-Fol) levels were measured at the Department of Clinical Biochemistry, Aarhus University Hospital, Denmark, employing the Advia Centaur CP Immunoassay System (Siemens) (P-Cob and P-Fol) and Liquid Chromatography–Tandem Mass Spectrometry on the AB SCIEX Triple Quad 5500 System (AB SCIEX) (P-MMA). The total imprecisions were 7.5% and 11.8%. Of these biomarkers, S-FE and S-TfR were used as biomarkers of iron status, S-RBP was used as a proxy marker of vitamin A status, P-Cob and P-MMA as biomarkers of cobalamin status, and P-Fol as a marker of folate status. S-CRP and S-AGP were the biomarkers of inflammation. We defined depleted iron stores if S-FE < 12 µg/L [21], tissue iron deficiency if S-TfR > 8.3 mg/L [20], low P-Cob if <148 pmol/L and marginal if 148–221 pmol/L [11], high P-MMA > 0.75 µmol/L [22], low P-Fol if <14 nmol/L [21], and low S-RBP if <0.7 µmol/L [21]. To estimate the prevalence of biomarkers indicating low iron and vitamin A levels, individual values of S-FE and S-RBP were corrected for inflammation using the regression correction described by Cichon et al. [23] and reported as S-FE corrected for inflammation and S-RBP corrected for inflammation. S-CRP > 5 mg/L and S-AGP > 1 g/L were used as cut-off values to define inflammation in the regression correction model. S-CRP cut-off values of 2 to <5, 5 to <10, 10 to 15, >15 mg/L and S-AGP 0.8 to 1.2, >1.2 g/L were used to adjust for inflammation when assessing correlates.

### 2.4. Statistical Analysis

Data were double-entered using EPIDATA 3.1 software (Epidata Association, Odense, Denmark), and double-entry quality checks were performed regularly. Anthropometric indices HAZ and WHZ were calculated using the World Health Organization (WHO) growth standards STATA igrowup package (WHO Anthro macro).

Background characteristics were summarized as percentages (%), mean (SD, standard deviation), or median [IQR, interquartile range]. A Pearson’s chi-square test was used to test for any difference in frequencies of categorical variables. Linear regression analysis was used to assess the potential correlates of the different micronutrient biomarkers while adjusting for age, sex, and inflammation (S-CRP and S-AGP). Non-normally distributed outcomes were log10-transformed for analysis, and the ratio of geometric means were interpreted as reflecting relative differences per unit increase in the exposure variables. Statistical significance was set at *p* < 0.05. All statistical analyses were carried out using STATA v15.1 (StataCorp, College Station, TX, USA).

### 2.5. Ethical Consideration

This study was conducted in accordance with the principles of the Declaration of Helsinki. Consent was obtained from the caregivers prior to inclusion. Verbal and written information was provided in Lusoga, Luganda, or English. Illiterate caregivers consented by giving a thumbprint in the presence of a literate witness. Permission to conduct the MAGNUS trial was approved by the School of Medicine Research Ethics Committee of Makerere University (#REC REF 2019-013) and the Ugandan National Council of Science and Technology (SS 49270). A consultative approval was given by the Danish National Committee on Biomedical Research Ethics (1906848). The MAGNUS trial is registered at www.isrctn.com (ISRCTN13093195).

## 3. Results

### 3.1. Participant Characteristics

Of 1112 children referred to the study sites after the initial screening, 831 were assessed, and 750 were eligible and enrolled in the study. The mean (SD) age was 32.0 (11.7) months, and 45.1% (*n* = 338) were girls. The mean HAZ was −3.02 (0.74), 41.9% (*n* = 314) were severely stunted (HAZ < −3), and 12.7% (*n* = 95) were still breastfed (Table 1). In the past 24 h prior, 30% of children (*n* = 227) had consumed dairy, 8.2% (62) meat, 30% (*n* = 227) fish, and 7.9% (*n* = 59) eggs. Of the 750 children enrolled, 55% (*n* = 413) were from the communities within the Buwenge health centre catchment area.

Data on micronutrient biomarkers were available for 92.4% to 98.8% of the children (Table 2). The prevalence of tissue iron deficiency (S-TfR > 8.3 mg/L) was 61.7% (*n* = 457), whilst that of depleted iron stores (S-Fe < 12 µg/L) increased from 16.6% to 42.9% with correction for inflammation. The prevalence of tissue iron deficiency was higher among children with depleted iron stores (77.7 vs. 49.7%, *p* < 0.001). Marginal or low P-Cob (<222 pmol/L) was found in 23.4% (*n* = 169) and elevated P-MMA (>0.75 µmol/L) in 15.8% (*n* = 116) of the children. The prevalence of elevated P-MMA was higher among those with P-Cob < 222 pmol/L than among those with P-Cob ≥ 222 pmol/L (28.9 vs. 11.7%, *p* < 0.001). Only 1.2% (*n* = 8) of the children had low folate (<14 nmol/L). Correction of S-RBP for inflammation reduced the prevalence of low vitamin A (S-RBP < 0.7 µmol/L) from 46.0% (*n* = 341) to 21.3% (*n* = 158).

### 3.2. Correlates of Markers of Iron Status

Correlates of S-FE and S-TfR are presented in Table 3. Girls compared to boys had 3.1 (95% CI 1.6, 4.5) mg/L lower S-TfR and marginally significantly higher S-FE (11%; 95% CI −1, 24) after adjusting for age and inflammation. Iron status was better in older children. Compared to children aged 12–23 months, children 24–35 and 36–59 months of age had 67 (95% CI 45, 92) % and 130 (95% CI 100, 164) % higher S-FE, respectively, and 3.0 (95% CI 1.2, 4.8) and 5.3 (95% CI 3.5, 7.1) mg/L lower S-TfR, respectively. Breastfed children had 18 (95% CI 0, 32) % lower S-FE and 5.3 (95% CI 2.8, 7.7) mg/L higher S-TfR. A positive malaria test was associated with higher S-FE and S-TfR levels. The magnitude of association was approximately halved after adjusting for inflammation, i.e., a positive malaria test was still associated with higher S-FE (75%; 95% CI 55, 98) and S-TfR (1.9 mg/L; 95% CI 0.2, 3.5). Further adjustment for maternal education and owning livestock did not change the estimates between breastfeeding and malaria and the markers of iron status. S-FE levels increased with increasing S-CRP and S-AGP levels. S-TfR was roughly 3 mg/L higher with S-CRP above 2 mg/L, with no dose–response relationship.

### 3.3. Correlates of Markers of Cobalamin Status

Girls had marginally higher P-Cob (17 pmol/L; 95% CI −2, 37) after adjustments for age and inflammation and, correspondingly, a 12 (95% CI 2, 21) % lower P-MMA (Table 4). Being breastfed was associated with marginally lower P-Cob (−29 pmol/L; 95% CI −63, 5) and 59 (95% CI 31, 92) % higher P-MMA. Additional adjustments for maternal education and owning livestock did not change the estimates between breastfeeding and markers of cobalamin status. After adjusting for inflammation, a positive malaria test was associated with 34 (95% CI 12, 57) pmol/L lower P-Cob and 33 (95% CI 18, 51) % higher P-MMA. Children with lower P-Fol (<20 nmol/L) had marginally significantly higher P-Cob (35 pmol/L; 95% CI 0, 71) and 24 (−95% CI 7, 37) % lower P-MMA compared to those having higher P-Fol > 30 nmol/L.

### 3.4. Correlates of Marker for Folate Status

Girls had higher P-Fol than boys (1.9 nmol/L; 95% CI 0.2, 3.6). Compared with children aged 12–23 months, children aged 24–35 months (2.1 nmol/L; 95% CI 0.0, 4.2) and 36–59 months (3.5 nmol/L; 95% CI 1.5, 5.6) had higher P-Fol (Table 5). Current breastfeeding was associated with a 4.5 (95% CI 1.6, 7.5) nmol/L higher P-Fol, and further adjustments for maternal education and owning livestock did not change the estimates.

### 3.5. Correlates of Marker for Vitamin A Status

Being currently breastfed was associated with 0.12 (95% CI 0.05, 0.19) µmol/L higher S-RBP (Table 5). A positive malaria test was associated with 0.05 (95% CI 0.00, 0.09) µmol/L lower S-RBP. However, after adjusting for inflammation, the malaria test was instead associated with 0.06 (95% CI 0.02, 0.11) µmol/L higher S-RBP. Further adjustments for maternal education and owning livestock did not change the estimates between breastfeeding and malaria and S-RBP. Elevated levels of S-CRP of >2 mg/L and S-AGP of >1.2 g/L were associated with lower S-RBP.

## 4. Discussion

Our study showed that approximately half of the children with stunting had iron deficiency, and one-fifth had low or marginal cobalamin or vitamin A deficiency. Sex, age, breastfeeding, malaria test, and inflammation were associated with micronutrient status.

The high levels of iron deficiency observed in this study are comparable to those reported in children in sub-Saharan Africa [8], where diets are often limited in bio-available iron and helminth infections are common. The prevalence of low or marginal P-Cob was relatively low in this study compared to other studies involving African children. Two-thirds of moderately malnourished Burkinabe children [24] and half of Kenyan schoolchildren [25] had low or marginal cobalamin. The differences in the burden of low or marginal cobalamin are likely due to the varying consumption of animal-source foods in the different study populations. In the current study, close to one-third of the children had consumed fish in the past 24 h, and fish can be a good source of cobalamin [26]. The prevalence of vitamin A deficiency in this study was higher (21%) than that previously reported in the general population of children under five years in Africa. Vitamin A deficiency was estimated at 8.9% (RBP < 0.8 µmol/L) in Uganda [27], 9.2% (RBP < 0.7 µmol/L) in Kenya [28], and 10% (RBP < 0.7 µmol/L) in Malawi [29]. Additionally, a cross-sectional study in Uganda found that low S-RBP (<0.8 µmol/L) was associated with higher odds of stunting in children aged 6–59 months [27]. This might be explained by the similar background environment of undernutrition and infections, where both stunting and vitamin A deficiency are prevalent and can co-exist in the same child. Very few children had biomarkers indicating low folate status, possibly because folate is bio-available from a wide range of food groups.

Older children (24–59 months) had better iron status; the observed lower iron status among younger children (12–23 months) is probably related to the increased iron requirements due to higher growth rates. Notably, iron requirements per kilogram of body weight are higher during the ages of 6–24 months [30].

Breastfeeding beyond the first year was associated with lower iron stores, more tissue iron deficiency, and lower cobalamin status. This may be related to the child’s overall food intake, as breastfeeding >1 year can potentially be associated with a preference for breastmilk over other foods, hence poor appetite for family foods [31]. In addition, impoverished mothers may decide to breastfeed longer due to lack of nutritious food to give their child. In the absence of adequate intake of quality complementary foods, breast milk is insufficient to meet the child’s iron and cobalamin requirements beyond infancy [32,33]. Conversely, breastfed children had higher P-Fol and S-RBP, as breast milk is probably an additional source of folate and vitamin A in their diets [32].

Interestingly, our study found a strong negative association between cobalamin status and malaria that was independent of inflammation. This is consistent with recently published data from moderately malnourished Burkinabe children, where a positive malaria test was associated with low P-Cob [24]. Yet, in this study we base the finding on not only P-Cob but also on P-MMA, which is the specific marker of cobalamin status. Cobalamin is a co-factor in methionine synthesis within the malarial parasite [34,35]. If the association between low cobalamin status and malaria reflects uptake of cobalamin by the malarial parasites, then malaria may be an important contributor to global cobalamin deficiency. Comparably, the fish tapeworm (*Dibothriocephalus latus*) has been reported to cause megaloblastic anaemia by competing with the host for cobalamin [36]. Alternatively, the lower P-Cob in children with positive malaria tests could be explained by higher cobalamin requirements due to rapid red cell production after haemolysis. A positive malaria test was also associated with biomarkers indicating greater iron stores but more tissue deficiency, which is partly explained by the presence of inflammation. Similarly, a recent review and meta-analysis found strong evidence that malaria infection, asymptomatic or symptomatic, is associated with increased S-FE concentrations in children [37]. Destruction of malaria-infected red blood cells, coupled with suppressed erythropoiesis and ferritin release from the damaged liver and spleen cells, results in increased S-Fe levels [38,39]. In contrast, the presence of immune or non-immune malaria-induced haemolysis stimulates erythropoiesis, thus increasing iron demand for red cell production and upregulating S-TfR [38]. A recently published study showed that malaria reduction interventions like increased coverage of insecticide-treated mosquito nets contributed to decline in childhood stunting in Uganda [40]. Such malaria reduction interventions can as well contribute to reducing some micronutrient deficiencies in stunted children.

As expected, increased levels of inflammatory biomarkers were associated with greater S-FE in a dose-dependent manner, higher S-TfR, and lower S-RBP. Similar findings have been reported based on pooled national survey cross-sectional data of children [41,42,43]. During the inflammatory response, iron is sequestered into its storage form (ferritin) as part of the body’s innate immune response to reduce the amount of iron available for pathogenic proliferation. S-RBP is a negative acute-phase protein and, in the presence of inflammation and infections, there is a reduced intake due to anorexia and reduced intestinal absorption of vitamin A [44]. In addition, there is decreased liver synthesis of S-RBP and its carrier protein and considerable retinol-RBP loss in urine. In this study, a lower cobalamin status was inconsistently associated with inflammation, as both cobalamin biomarkers were associated with either very high S-CRP (>15 mg/L) or S-AGP. Previously published data from 16 national surveys found a weak and inconsistent correlation between S-CRP or S-AGP and cobalamin or folate biomarkers [45].

We did not find increased P-Fol in children with low P-Cob, as expected from the methylTHF trap hypothesis [46], probably because few children had very low P-Cob. Likewise, it was surprising that P-Fol < 20 nmol/L was associated with higher P-Cob, since low folate has been reported to be accompanied by low cobalamin concentration, although the mechanism is not understood [46].

The strength of this study is its ability to assess multiple micronutrient biomarkers in a vulnerable population of stunted children. Furthermore, we had a large sample size with a near complete data set of micronutrient biomarkers and recruited children from the community. A major limitation of this study is the cross-sectional design, which can only suggest associations. Second, for comparison, we had no children without stunting. Third, the cut-off values for P-Cob and P-MMA used to estimate the prevalence did not take age into account. Universally agreed age-specific P-Cob and P-MMA were not available. Fourth, we only measured plasma folate and not red blood cell folate, which is a maker of more long-term folate intake and status. Additionally, we did not measure serum retinol in a sub-sample population and, therefore, could not generate population-specific S-RBP cut-offs. However, we used the recommended serum retinol cut-off value <0.7 µmol/L and assumed a 1:1 ratio with S-RBP, in addition to correcting S-RBP for inflammation before estimating prevalence. Lastly, our findings may have limited generalizability since we only recruited from two communities in eastern Uganda.

In conclusion, a large proportion of children with stunting in a low-resource setting have iron, cobalamin, or vitamin A deficiency. Interventions addressing stunting should also target improving existing micronutrient deficiencies. Breastfeeding beyond the first year and malaria are associated with tissue iron deficiency and lower cobalamin status. The strong negative association between cobalamin status and malaria warrants further research.

## Figures and Tables

**Table 1:** Background characteristics of 750 children aged 12 to 59 months with stunting.

Characteristic	Value	N
Age, months	32 ±11.7	750
12–23	30% (222)	
24–35	34% (259)	
36–59	36% (269)	
Sex, girls	45% (338)	750
Residence, urban	55% (415)	750
Household size	5 [4; 7]	750
Maternal education, primary and above	54% (399)	737
Own livestock	51% (379)	750
Breastfed, currently	13% (95)	746
Height-for-age, z-score	−3.02 ±0.74	750
<−3	42% (314)	
Weight-for-height, z-score	−0.36 ±0.99	749
Mid-upper arm circumference, mm	14.4 ±1.18	750
Serum C-reactive protein, mg/L	1.57 [0.33; 8.25]	741
<2	53% (396)	
2–<5	13% (94)	
5–<10	12% (88)	
10–15	5% (35)	
>15	17% (128)	
Serum α_1_-acid glycoprotein, g/L	1.29 ±0.52	741
<0.8	19% (139)	
0.8–1.2	31% (232)	
>1.2	50% (370)	
Malaria rapid test positive	40% (292)	737

Data are mean ±standard deviation, median [interquartile range], or % (frequency, *n*).

**Table 2:** Biomarkers of micronutrient status of 750 children with stunting ^1^.

Micronutrient Biomarker	N	Uncorrected	Inflammation Corrected ^2^
Serum ferritin, µg/L	741	37.7 [16.9; 78.7]	13.6 [7.6; 22.9]
<12		16.6% (123)	42.9% (318)
Serum soluble transferrin receptor, mg/L	741	14.6 ±10.4	-
>8.3		61.7% (457)	-
Plasma cobalamin, pmol/L	719	316 ±133	-
<148		3.5% (25)	-
148–221		20.0% (144)	-
Plasma methylmalonic acid, µmol/L	733	0.32 [0.20; 0.55]	-
>0.75		15.8% (116)	-
Plasma folate, nmol/L	692	34.7 ±11.2	-
<14		1.2% (8)	-
Serum retinol binding protein, µmol/L	741	0.76 ±0.29	0.89 ±0.27
<0.7		46.0% (341)	21.3% (158)

^1^ Values are presented as *n* number of available samples, median [interquartile range], mean ±standard deviation, or % (number, n); ^2^ Corrected for inflammation by regression method using both C-reactive protein > 5 mg/L and α_1_-acid glycoprotein > 1 g/L.

**Table 3:** Correlates of biomarkers for iron status among children aged 12 to 59 months with stunting ^1^.

	Serum Ferritin, % ^2^ (N = 741)					Serum Soluble Transferrin Receptor, mg/L (N = 741)				
	n	Model 1 ^‡^ 10 ^β^ (95% CI)	*p*	Model 2 ^§^ 10 ^β^ (95% CI)	*p*	n	Model 1 ^‡^ β (95% CI)	*p*	Model 2 ^§^ β (95% CI)	*p*
Sex										
Boy	407	-	-	-	-	407	-	-	-	-
Girls	334	3 (−10; 19)	0.65	11 (−1; 24)	0.06	334	−3.3 (−4.8; −1.8)	<0.001	−3.1 (−4.5; −1.6)	<0.001
Age, months										
12–23	218	-	-	-	-	218	-	-	-	-
24–35	256	78 (50; 114)	<0.001	67 (45; 92)	<0.001	256	−2.9 (−4.7; −1.1)	0.002	−3.0 (−4.8; −1.2)	0.001
36–59	267	143 (103; 188)	<0.001	130 (100; 164)	<0.001	267	−5.3 (−7.1; −3.5)	<0.001	−5.3 (−7.1; −3.5)	<0.001
Stunting degree										
Moderate	430	-	-	-	-	430	-	-	-	-
Severe	311	18 (3; 37)	0.02	2 (−9; 15)	0.75	311	0.3 (−1.2; 1.8)	0.70	−0.2 (−1.7; 1.3)	0.84
Breastfed currently										
No	643	-	-	-	-	643	-	-	-	-
Yes	94	−24 (−41; −3.3)	0.02	−18 (−32; 0)	0.05	94	5.1 (2.6; 7.5)	<0.001	5.3 (2.8; 7.7)	<0.001
Malaria rapid test										
Negative	439	-	-	-	-	439	-	-	-	-
Positive	291	167 (134; 204)	<0.001	75 (55; 98)	<0.001	291	2.8 (1.3; 4.3)	<0.001	1.9 (0.2; 3.5)	0.02
Serum C-reactive protein, mg/L										
<2	396	-	-			396	-	-		
2–<5	94	73 (44; 108)	<0.001			94	3.9 (1.6; 6.1)	0.001		
5–<10	88	124 (86; 171)	<0.001			88	2.4 (0.1; 4.7)	0.04		
10–15	35	137 (79; 215)	<0.001			35	3.0 (−0.5; 6.4)	0.09		
>15	128	322 (259; 397)	<0.001			128	3.0 (1.0; 5.0)	0.003		
Serum α_1_-acid glycoprotein, g/L										
<0.8	139	-	-			139	-	-		
0.8–1.2	232	36 (14; 62)	0.001			232	1.1 (−1.0; 3.2)	0.31		
>1.2	370	224 (97: 282)	<0.001			370	3.3 (1.4; 5.3)	0.001		

^1^ Data are number, percentage (ratio of geometric means) or mean difference, and 95% confidence interval. ^2^ Log-transformed, the back-transformed regression coefficient 10 ^β^ reflects percentage change in serum ferritin per 1-unit increase in the correlates. ^‡^ Model 1 Linear regression analysis adjusting for age and sex. Age was adjusted for sex, and vice versa. ^§^ Model 2 Linear regression analysis adjusting for age, sex, and inflammation. Serum C-reactive protein and serum α_1_-acid glycoprotein as categorical variables were used to adjust for inflammation, but mutually adjusted coefficients are not shown.

**Table 4:** Correlates of biomarkers for cobalamin status among children aged 12 to 59 months with stunting ^1^.

	Plasma Cobalamin, pmol/L (N = 719)					Plasma Methylmalonic Acid, % ^2^ (N = 733)				
	n	Model 1 ^‡^ β (95% CI)	*p*	Model 2 ^§^ β (95% CI)	*p*	n	Model 1 ^‡^ 10 ^β^ (95% CI)	*p*	Model 2 ^§^ 10 ^β^ (95% CI)	*p*
Sex										
Boy	392	-	-	-	-	400	-	-	-	-
Girls	327	18 (−1; 38)	0.06	17 (−2; 37)	0.08	333	−13 (−22; −4)	0.008	−12 (−21; −2)	0.01
Age, months										
12–23	208	-	-	-	-	213	-	-	-	-
24–35	246	−16 (−40; 9)	0.21	−14 (−38; 10)	0.24	252	−9 (−20; 4)	0.18	−9 (−20; 5)	0.19
36–59	265	−1 (−25; 23)	0.94	−1 (−25; 23)	0.95	268	−9 (−20; 4)	0.18	−7 (−18; 7)	0.31
Stunting degree										
Moderate	415	-	-	-	-	-	-	-	-	-
Severe	304	6 (−14; 25)	0.53	7 (−14; 27)	0.51	306	5 (−6; 17)	0.35	1 (−9; 13)	0.80
Breastfed currently										
No	626	-	-	-	-	640	-	-	-	-
Yes	89	−24 (−58; 10)	0.17	−29 (−63; 5)	0.09	89	49 (24; 80)	<0.001	59 (31; 92)	<0.001
Malaria rapid test										
Negative	430	-	-	-	-	438	-	-	-	-
Positive	279	−41 (−61; −21)	<0.001	−34 (−57; −12)	0.003	285	31 (17; 47)	<0.001	33 (18; 51)	<0.001
Serum C-reactive protein, mg/L										
<2	379	-	-			388	-	-		
2–<5	92	1 (−29; 31)	0.94			94	5 (−11; 23)	0.60		
5–<10	85	−13 (−44; 18)	0.40			86	−1 (−17; 17)	0.87		
10–15	32	−7 (−55; 40)	0.76			32	−3 (−26; 26)	0.80		
>15	125	−44 (−71; −18)	0.001			127	2 (−12; 18)	0.79		
Serum α_1_-acid glycoprotein, g/L										
<0.8	135	-	-			137	-	-		
0.8–1.2	224	9 (−21; 35)	0.63			229	16 (−1; 30)	0.01		
>1.2	354	−8 (−34; 18)	0.56			361	24 (8; 44)	0.003		
Serum folate, nmol/L										
>30	422	-	-	-	-	422	-	-	-	-
20–30	202	−3 (−26; 19)	0.77	0 (−23; 22)	0.98	202	−1 (−12; 12)	0.87	−4 (−15; 8)	0.50
<20	62	29 (−6; 66)	0.10	35 (0; 71)	0.05	62	−21 (−35; −4)	0.01	−24 (−37; −7)	0.007

^1^ Data are number, mean difference or percentage (ratio of geometric means), and 95% confidence interval. ^2^ Log-transformed, the back-transformed regression coefficient 10 ^β^ reflects percentage change in plasma methylmalonic acid per 1-unit increase in the correlates. ^‡^ Model 1 Linear regression analysis adjusting for age and sex. Age was adjusted for sex, and vice versa. ^§^ Model 2 Linear regression analysis adjusting for age, sex, and inflammation. Serum C-reactive protein and α_1_-acid glycoprotein as categorical variables were used to adjust for inflammation, but mutually adjusted coefficients are not shown.

**Table 5:** Correlates of biomarkers of folate and retinol status among children aged 12 to 59 months with stunting ^1^.

	Plasma Folate, nmol/L (N = 692)					Serum Retinol Binding Protein, µmol/L (N = 741)				
	n	Model 1 ^‡^ β (95% CI)	*p*	Model 2 ^§^ β (95% CI)	*p*	n	Model 1 ^‡^ β (95% CI)	*p*	Model 2 ^§^ β (95% CI)	*p*
Sex										
Boy	383	-	-	-	-	407	-	-	-	
Girls	309	2.2 (0.5; 3.9)	0.01	1.9 (0.2; 3.6)	0.02	334	0.02 (−0.02; 0.06)	0.32	0.01 (−0.03; 0.05)	0.657
Age, months										
12–23	199	-	-	-	-	218	-	-	-	-
24–35	231	1.9 (−0.2; 4.0)	0.07	2.1 (0.0; 4.2)	0.04	256	−0.03 (−0.08; 0.03)	0.32	−0.02 (−0.07; 0.03)	0.43
36–59	262	3.5 (1.5; 5.6)	0.001	3.5 (1.5; 5.6)	0.001	267	−0.05 (−0.10; 0.01)	0.08	−0.04 (−0.08; 0.01)	0.14
Stunting degree										
Moderate	401	-	-	-	-	430	-	-	-	-
Severe	291	0.0 (−1.7; 1.6)	0.96	0.6 (−1.1; 2.4)	0.47	311	−0.01 (−0.06; 0.03)	0.545	0.01 (−0.03; 0.05)	0.775
Breastfed currently										
No	605	-	-	-	-	643				
Yes	83	4.7 (1.7; 7.6)	0.002	4.5 (1.6; 7.5)	0.003	94	0.13 (0.05; 0.20)	0.001	0.12 (0.05; 0.19)	0.001
Malaria rapid test										
Negative	416	-	-	-	-	439	-	-	-	-
Positive	266	−2.2 (−3.9; −0.5)	0.01	−0.8 (−2.7; 1.1)	0.41	291	−0.05 (−0.09; 0.00)	0.03	0.06 (0.02; 0.11)	0.004
Serum C-reactive protein, mg/L										
<2	369	-	-			396	-	-		
2–<5	87	−2.7 (−5.3; −0.1)	0.04			94	−0.10 (−0.16; −0.04)	0.001		
5–<10	80	−2.5 (−5.2; 0.2)	0.06			88	−0.07 (−0.13; −0.01)	0.02		
10–15	32	−1.8 (−5.8; 2.2)	0.38			35	−0.25 (−0.34; −0.16)	<0.001		
>15	119	−3.67 (−6.0; −1.4)	0.002			128	−0.29 (−0.35; −0.24)	<0.001		
Serum α_1_-acid glycoprotein, g/L										
<0.8	136	-	-			139	-	-		
0.8–1.2	219	−2.3 (−4.7; 0.0)	0.05			232	−0.02 (−0.07; 0.04)	0.61		
>1.2	332	−4.6 (−6.8; −2.4)	<0.001			370	−0.16 (−0.22; −0.11)	<0.001		
Plasma cobalamin, pmol/L										
>221	530	-	-	-	-					
148–221	138	1.0 (−1.1, 3.1)	0.35	1.2 (−0.9, 3.3)	0.25					
<148	24	3.6 (−1.0, 8.2)	0.12	3.3 (−1.2, 7.9)	0.15					

^1^ Data are number, mean difference, and 95% confidence interval. ^‡^ Model 1 Linear regression analysis adjusting for age and sex. Age was adjusted for sex, and vice versa. ^§^ Model 2 Linear regression analysis adjusting for age, sex, and inflammation. Serum C-reactive protein and α_1_-acid glycoprotein as categorical variables were used to adjust for inflammation, but mutually adjusted coefficients are not shown.

## Data Availability

The Ugandan act on Data Protection and Privacy and the European act on General Data Protection Regulation do not allow for personal data to be made available to other researchers without prior written approval from relevant institutions and authorities. For further information, please contact the corresponding author.

## References

[1] World Health Organization, Food Agriculture Organization of the United Nations (2004). Vitamin and Mineral Requirements in Human Nutrition.

[2] Stevens G.A., Beal T., Mbuya M.N.N., Luo H., Neufeld L.M., Addo O.Y., Adu-Afarwuah S., Alayón S., Bhutta Z., Brown K.H. (2022). Micronutrient deficiencies among preschool-aged children and women of reproductive age worldwide: A pooled analysis of individual-level data from population-representative surveys. Lancet Glob. Health.

[3] Balarajan Y., Ramakrishnan U., Özaltin E., Shankar A.H., Subramanian S. (2011). Anaemia in low-income and middle-income countries. Lancet.

[4] Black M.M. (2008). Effects of Vitamin B_12_ and Folate Deficiency on Brain Development in Children. Food Nutr. Bull..

[5] McCann J.C., Ames B.N. (2007). An overview of evidence for a causal relation between iron deficiency during development and deficits in cognitive or behavioral function. Am. J. Clin. Nutr..

[6] Prado E.L., Dewey K.G. (2014). Nutrition and brain development in early life. Nutr. Rev..

[7] Mayo-Wilson E., Imdad A., Herzer K., Yakoob M.Y., Bhutta Z.A. (2011). Vitamin A supplements for preventing mortality, illness, and blindness in children aged under 5: Systematic review and meta-analysis. BMJ.

[8] Muriuki J.M., Mentzer A.J., Webb E.L., Morovat A., Kimita W., Ndungu F.M., Macharia A.W., Crane R.J., Berkley J.A., Lule S.A. (2020). Estimating the burden of iron deficiency among African children. BMC Med..

[9] Stevens G.A., Bennett J.E., Hennocq Q., Lu Y., De-Regil L.M., Rogers L., Danaei G., Li G., White R.A., Flaxman S.R. (2015). Trends and mortality effects of vitamin A deficiency in children in 138 low-income and middle-income countries between 1991 and 2013: A pooled analysis of population-based surveys. Lancet Glob. Health.

[10] Allen L.H., Miller J.W., de Groot L., Rosenberg I.H., Smith A.D., Refsum H., Raiten D.J. (2018). Biomarkers of Nutrition for Development (BOND): Vitamin B-12 Review. J. Nutr..

[11] World Health Organization, United Nations Children’s Fund (2021). The World Bank. Levels and Trends in Child Malnutrition: UNICEF/WHO/The World Bank Group Joint Child Malnutrition Estimates: Key Findings of the 2021 Edition.

[12] Prendergast A.J., Humphrey J.H. (2014). The stunting syndrome in developing countries. Paediatr. Int. Child Health.

[13] Miller V., Reedy J., Cudhea F., Zhang J., Shi P., Erndt-Marino J., Coates J., Micha R., Webb P., Mozaffarian D. (2022). Global, regional, and national consumption of animal-source foods between 1990 and 2018: Findings from the Global Dietary Database. Lancet Planet. Health.

[14] Suchdev P.S., Namaste S.M., Aaron G.J., Raiten D.J., Brown K.H., Flores-Ayala R. (2016). Overview of the Biomarkers Reflecting Inflammation and Nutritional Determinants of Anemia (BRINDA) Project. Adv. Nutr..

[15] Darnton-Hill I., Ahmed F., Bendich A., Deckelbaum R.J. (2010). Micronutrients: Immunological and infection effects on nutritional status and impact on health in developing countries. Preventive Nutrition: The Comprehensive Guide for Health Professionals.

[16] Victora C.G., Adair L., Fall C., Hallal P.C., Martorell R., Richter L., Sachdev H.S., Maternal and Child Undernutrition Study Group (2008). Maternal and child undernutrition: Consequences for adult health and human capital. Lancet.

[17] Victora C.G., de Onis M., Hallal P.C., Blössner M., Shrimpton R. (2010). Worldwide Timing of Growth Faltering: Revisiting Implications for Interventions. Pediatrics.

[18] Pesu H., Mutumba R., Mbabazi J., Olsen M.F., Mølgaard C., Michaelsen K.F., Ritz C., Filteau S., Briend A., Mupere E. (2021). The Role of Milk Protein and Whey Permeate in Lipid-based Nutrient Supplements on the Growth and Development of Stunted Children in Uganda: A Randomized Trial Protocol (MAGNUS). Curr. Dev. Nutr..

[19] Uganda Bureau of Statistcs (UBOS) and ICF (2017). Uganda Demographic and Health Survey 2016: Key Indicators Report.

[20] Erhardt J.G., Estes J.E., Pfeiffer C.M., Biesalski H.K., Craft N.E. (2004). Combined Measurement of Ferritin, Soluble Transferrin Receptor, Retinol Binding Protein, and C-Reactive Protein by an Inexpensive, Sensitive, and Simple Sandwich Enzyme-Linked Immunosorbent Assay Technique. J. Nutr..

[21] Centers for Disease Control and Prevention, World Health Organization, Nutrition International, UNICEF (2020). Micronutrient Survey Manual.

[22] Heil S.G., de Jonge R., de Rotte M.C.F.J., van Wijnen M., Heiner-Fokkema R.M.R., Kobold A.C.M., Pekelharing J.M.M., Adriaansen H.J., Sanders E., Trienekens P.H. (2012). Screening for metabolic vitamin B_12_ deficiency by holotranscobalamin in patients suspected of vitamin B_12_ deficiency: A multicentre study. Ann. Clin. Biochem..

[23] Cichon B., Ritz C., Fabiansen C., Christensen V.B., Filteau S., Friis H., Kæstel P. (2017). Assessment of Regression Models for Adjustment of Iron Status Biomarkers for Inflammation in Children with Moderate Acute Malnutrition in Burkina Faso. J. Nutr..

[24] Friis H., Cichon B., Fabiansen C., Iuel-Brockdorff A.-S., Yaméogo C.W., Ritz C., Frikke-Schmidt R., Briend A., Michaelsen K.F., Christensen V.B. (2022). Serum cobalamin in children with moderate acute malnutrition in Burkina Faso: Secondary analysis of a randomized trial. PLoS Med..

[25] McLean E.D., Allen L.H., Neumann C.G., Peerson J.M., Siekmann J.H., Murphy S.P., Bwibo N.O., Demment M.W. (2007). Low Plasma Vitamin B-12 in Kenyan School Children Is Highly Prevalent and Improved by Supplemental Animal Source Foods. J. Nutr..

[26] Nölle N., Genschick S., Schwadorf K., Hrenn H., Brandner S., Biesalski H.K. (2020). Fish as a source of (micro)nutrients to combat hidden hunger in Zambia. Food Secur..

[27] Ssentongo P., Ba D.M., Ssentongo A.E., Fronterre C., Whalen A., Yang Y., Ericson J.E., Chinchilli V.M. (2020). Association of vitamin A deficiency with early childhood stunting in Uganda: A population-based cross-sectional study. PLoS ONE.

[28] Shadrack O., Ngowa S., Joseph A., Carolyne A., Benzadze N., Mbesa K., Joseph O., Oscar K., Rachel K., Richard M. (2019). Programmatic implications of some vitamin A supplementation and deworming determinants among children aged 6–59 months in resource-poor rural Kenya. Pan. Afr. Med. J..

[29] Likoswe B.H., Joy E.J.M., Sandalinas F., Filteau S., Maleta K., Phuka J.C. (2021). Re-Defining the Population-Specific Cut-Off Mark for Vitamin A Deficiency in Pre-School Children of Malawi. Nutrients.

[30] Domellöf M., Braegger C., Campoy C., Colomb V., Decsi T., Fewtrell M., Hojsak I., Mihatsch W., Molgaard C., Shamir R. (2014). Iron Requirements of Infants and Toddlers. J. Pediatr. Gastroenterol. Nutr..

[31] Lackey K.A., Fehrenkamp B.D., Pace R.M., Williams J.E., Meehan C.L., McGuire M.A., McGuire M.K. (2021). Breastfeeding Beyond 12 Months: Is There Evidence for Health Impacts?. Annu. Rev. Nutr..

[32] Dror D.K., Allen L.H. (2018). Overview of Nutrients in Human Milk. Adv. Nutr. Int. Rev. J..

[33] Greibe E., Lildballe D.L., Streym S., Vestergaard P., Rejnmark L., Mosekilde L., Nexo E. (2013). Cobalamin and haptocorrin in human milk and cobalamin-related variables in mother and child: A 9-mo longitudinal study. Am. J. Clin. Nutr..

[34] Krungkrai J., Webster H.K., Yuthavong Y. (1989). Characterization of cobalamin-dependent methionine synthase purified from the human malarial parasite, Plasmodium falciparum. Parasitol. Res..

[35] Musabyimana J.P., Distler U., Sassmannshausen J., Berks C., Manti J., Bennink S., Blaschke L., Burda P.-C., Flammersfeld A., Tenzer S. (2022). *Plasmodium falciparum* S-Adenosylmethionine Synthetase Is Essential for Parasite Survival through a Complex Interaction Network with Cytoplasmic and Nuclear Proteins. Microorganisms.

[36] Scholz T.S., Garcia H.H., Kuchta R., Wicht B. (2009). Update on the human broad tapeworm (genus Diphyllobothrium), including clinical relevance. Clin. Microbiol. Rev..

[37] Sandalinas F., Filteau S., Joy E.J.M., de la Revilla L.S., MacDougall A., Hopkins H. (2022). Measuring the impact of malaria infection on indicators of iron and vitamin A status: A systematic literature review and meta-analysis. Br. J. Nutr..

[38] Lynch S., Pfeiffer C.M., Georgieff M.K., Brittenham G., Fairweather-Tait S., Hurrell R.F., McArdle H.J., Raiten D.J. (2018). Biomarkers of Nutrition for Development (BOND)—Iron Review. J. Nutr..

[39] Moya-Alvarez V., Bodeau-Livinec F., Cot M. (2016). Iron and malaria: A dangerous liaison?. Nutr. Rev..

[40] Keats E.C., Kajjura R.B., Ataullahjan A., Islam M., Cheng B., Somaskandan A., Charbonneau K.D., Confreda E., Jardine R., Oh C. (2022). Malaria reduction drives childhood stunting decline in Uganda: A mixed-methods country case study. Am. J. Clin. Nutr..

[41] Namaste S.M., Rohner F., Huang J., Bhushan N.L., Flores-Ayala R., Kupka R., Mei Z., Rawat R., Williams A.M., Raiten D.J. (2017). Adjusting ferritin concentrations for inflammation: Biomarkers Reflecting Inflammation and Nutritional Determinants of Anemia (BRINDA) project. Am. J. Clin. Nutr..

[42] Rohner F., Namaste S.M., Larson L.M., Addo O.Y., Mei Z., Suchdev P.S., Williams A.M., Ashour F.A.S., Rawat R., Raiten D.J. (2017). Adjusting soluble transferrin receptor concentrations for inflammation: Biomarkers Reflecting Inflammation and Nutritional Determinants of Anemia (BRINDA) project. Am. J. Clin. Nutr..

[43] Larson L.M., Guo J., Williams A.M., Young M.F., Ismaily S., Addo O.Y., Thurnham D., Tanumihardjo S.A., Suchdev P.S., Northrop-Clewes C.A. (2018). Approaches to Assess Vitamin A Status in Settings of Inflammation: Biomarkers Reflecting Inflammation and Nutritional Determinants of Anemia (BRINDA) Project. Nutrients.

[44] Rubin L.P., Ross A.C., Stephensen C.B., Bohn T., A Tanumihardjo S. (2017). Metabolic Effects of Inflammation on Vitamin A and Carotenoids in Humans and Animal Models. Adv. Nutr. Int. Rev. J..

[45] Young M.F., Guo J., Williams A., Whitfield K.C., Nasrin S., Kancherla V., Suchdev P.S., Crider K.S., Pfeiffer C.M., Serdula M. (2020). Interpretation of vitamin B-12 and folate concentrations in population-based surveys does not require adjustment for inflammation: Biomarkers reflecting inflammation and nutritional determinants of anemia (BRINDA) project. Am. J. Clin. Nutr..

[46] Carmel R., Ross A.C. (2014). Chapter 27: Cobalamin (Vitamin B12). Modern Nutrition in Health and Disease.

